# A case of *Vibrio vulnificus* infection complicated with fulminant purpura: gene and biotype analysis of the pathogen

**DOI:** 10.1099/jmmcr.0.005096

**Published:** 2017-05-19

**Authors:** Masatoshi Hori, Akifumi Nakayama, Daisuke Kitagawa, Hidetada Fukushima, Hideki Asai, Yasuyuki Kawai, Kazuo Okuchi

**Affiliations:** ^1^​ Department of Emergency and Critical Care Medicine, Nara Medical University, 840 Shijo-Cho, Kashihara, Nara, Japan; ^2^​ Department of Medical Technology, School of Health Sciences, Gifu University of Medical Science, 795-1 Aza-Nagamine, Ichihiraga, Seki City, Seki, Gifu, Japan; ^3^​ Department of Central Laboratory Medicine, Nara Prefecture General Medical Center, Hiramatsu, Nara, Nara Prefecture 631-0846, Japan

**Keywords:** *Vibrio vulnificus*, primary septicemia, fulminant purpura, biotype 3, genotype E

## Abstract

**Introduction.** *Vibrio vulnificus* (*V. vulnificus*) causes a severe infection that develops in the compromised host. Its pathophysiology is classified into three types: (1) primary septicaemia, (2) gastrointestinal illness pattern and (3) wound infection pattern. Of these, primary septicaemia is critical. *V. vulnificus* can be classified into three biotypes and two genotypes and its pathogenicity is type-dependent.

**Case presentation.** A 47-year-old man presented to a local hospital with chief complaints of fever, bilateral lower limb pain and diarrhoea. He had no history of foreign travel or known medical problems. He was in septic shock and developed fulminant purpura within 24 h of the onset. High-dose vasopressor and antibiotic administration failed to alter his status and he died 3 days after the onset of symptoms. *V. vulnificus* was isolated from blood, skin and nasal discharge cultures. Biotype and gene analysis of the microbe isolated identified it as Biotype 3, mainly reported in Israel in wound infections, and Genotype E, implicating an environmental isolate. These typing analyses indicated that the microbe isolated could be classified as a type with low pathogenicity.

**Conclusion.** This case highlighted that Biotype 3 and Genotype E can also cause primary septicaemia. Although the majority of reports on Biotype 3 have been from the Middle East, this experience with the present case provided evidence that the habitat of Biotype 3 *V. vulnificus* has been extending to East Asia as well.

## Introduction

*Vibrio vulnificus* is a Gram-negative, facultatively anaerobic rod distributed in warm, brackish water areas, and is known to cause severe infectious disease in the compromised host such as those with liver disorders or diabetes mellitus. Infection routes of *V. vulnificus* are oral and wound infection. Its pathophysiology is classified into three types: (1) primary septicaemia, (2) gastrointestinal illness pattern and (3) wound infection pattern. Primary septicaemia is caused by oral route infection with ingestion of raw seafood and results in a systemic infection with necrotizing fasciitis within 48 h [1–3]. In Japan, the majority of cases of *V. vulnificus* infection manifest as primary septicaemia, with a mortality rate of up to 70 %, and more than one-half of cases die within 3 days [4]. In this report, we present a case of primary septicaemia with fulminant purpura due to infection by *V. vulnificus* classified as low pathogenicity type by biotype and genotype analysis.

## Case report

A 47-year-old man with a history of hypertension and cholecystolithiasis was brought to the emergency department of a local hospital because of fever over 38 °C, bilateral lower limb pain and diarrhoea. He was a carpenter and was a habitual alcohol drinker (120 g per day) and smoker. One day prior to the presentation, he had a slight fever and anorexia. He was unconscious on arrival with Glasgow Coma Scale of E3V4M6 and blood pressure of 85/45 mmHg, and a heart rate of 110 beats per minute. He was intubated, and vasopressors were administered intravenously. Under a diagnosis of septic shock, 1 g doripenem was administered. Twelve hours after the presentation, erythema spread to the trunk and purpura developed on the lower limbs. Three hours later, purpura appeared on the face and trunk as well and he was transferred to our hospital because of persistent circulatory failure. Although the details of his dietary habits were unknown, he resided in an inland area and had no contact with seawater or history of foreign travel.

**Fig. 1.**
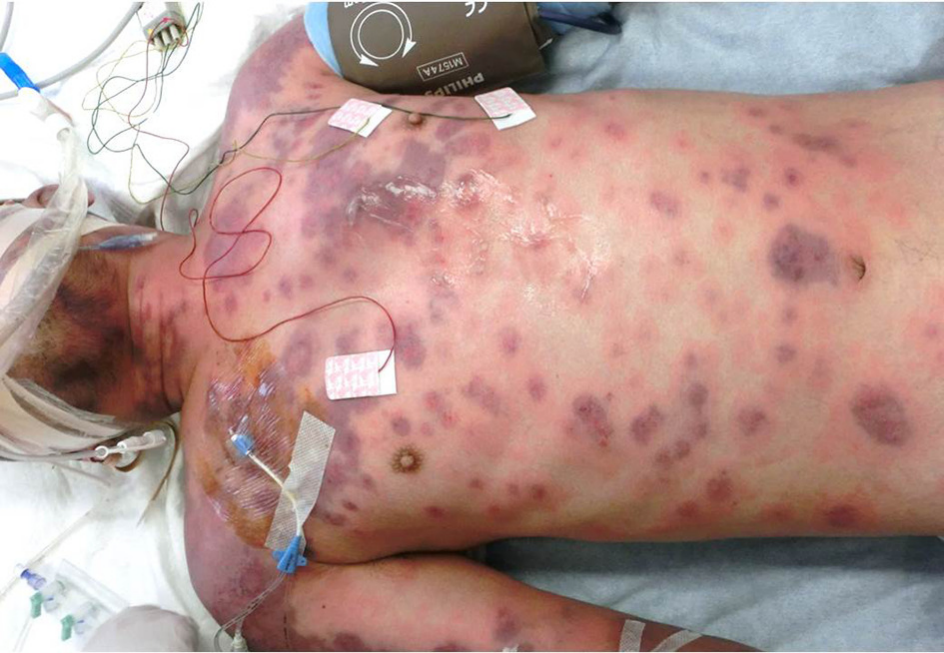
External surface systemic purpura surrounded by reddish areas was found, and serous and blood blistering was disseminated.

On arrival at our hospital, his Glasgow Coma Scale was E1VTM1, he had a spontaneous respiratory rate of 35 breaths per min and his blood pressure was 80/40 mmHg with continuous intravenous infusion of 1.05 µg noradrenaline kg^−1^ min^−1^. His body temperature was high, up to 40.5 °C. No abnormalities were noted on thoracic auscultation or abdominal palpation. On the external surface of his body, there were no visible scars, but severe oedema and systemic purpura were seen (Fig. 1). All of the purpuras were surrounded by reddish zones, and there was no desquamation, epidermis exfoliation or Nicolski’s sign.

## Investigations

Computed tomography of the abdomen showed hepatomegaly with no obvious signs of liver cirrhosis. Blood laboratory tests revealed decreased white blood cells and platelets with C-reactive protein of 21.2 mg dl^−1^. Plasma endotoxin level was markedly elevated to 4850 pg ml^−1^. *V. vulnificus* was isolated from blood, blister and nasal discharge cultures.

## Treatment

Under a diagnosis of *V. vulnificus*-induced septic shock with fulminant purpura, doripenem administration every 8 h, intravenous immunoglobulin, hydrocortisone and continuous renal replacement therapy were performed, but the patient remained unresponsive.

## Outcome and follow-up

The patient died on the third hospital day, and autopsy study conducted 4 h after death revealed subcutaneous blisters, necrotizing fasciitis and liver cirrhosis. *V. vulnificus* was not isolated from cultures of muscle, liver, gastric juice, small intestinal mucosa, stool, ascites or pleural effusion, but was isolated from the colonic mucosa and skin.

As shown in Table 1, the microbe isolated was classified as Biotype 3 from its biochemical properties. We performed a comparison of the present microbe with previously reported human-derived *V. vulnificus* CMCP6 [5]. Genomic DNA was purified using a NucleoSpin Tissue kit (Takara), and libraries were prepared from purified genomic DNA using a TruSeq Nano DNA Library Prep kit (Illumina). Analysis was then performed with a HiSeq next-generation sequencer (Illumina) by paired-end sequencing of 100 base pairs. These output data were analysed with the gene analysis server Takeru Lite for Sequencer IV (NABE International) for *de novo* assembly with MIRA v3.4 software. The assembled data (contig sequences) were uploaded to the RAST (Rapid Annotation using Subsystem Technology; http://rast.nmpdr.org/) server for annotation and functional characterization based on comparison with the *V. vulnificus* CMCP6 genome. Also, using Genetyx ver.11 (GENETYX), the primer sequence shown by Rosche *et al.* [6] was searched for based on homology in the genomic sequence of the microbe. As a result, a 277 base pair sequence amplified by an E-type primer set was found. Moreover, from the results of the function-based comparison of the annotated data, 18 types of known pathogenic factor were held in common with the genomic sequence of *V. vulnificus* CMCP6 (Table 2).

**Table 1.** Result of biotyping

Test	Biotype			Present microbe
	1	2	3	
Oxidase	+	+	+	+
Lysine decarboxylase	+	+	+	+
Sucrose fermentation	−	−	−	−
Ornithine decarboxylase	+	−	+	+
Indole production	+	−	+	+
d-Mannitol fermentation	+	−	−	−
d-Sorbitol fermentation	−	+	−	−
Citrate (Simmon’s)	+	+	−	−

**Table 2.** Pathogenic factors commonly present in *V. vulnificus* CMCP6 and the present microbe

No.	Pathogenic factor	Author
1	Arylsulfatase	Koton *et al*. [9]
2	Methyl-accepting chemotaxis protein	
3	Acetyltransferase	
4	GGDEF family protein	
5	Type VI secretion systems	
6	Integrase	
7	Protein secretion system, Type II	Hwang *et al.* [10]
8	HlyU (small metal-regulatory transcriptional repressor)	Liu *et al.* [11]
9	Thermolysin-like metalloproteases	Miyoshi [12]
10	Phospholipase A	Koo *et al.* [13]
11	Flagellin C	Kim *et al.* [14]
12	Flagellin D	
13	Flagella E	Lee *et al.* [15]
14	Capsular polysaccharide	Powell *et al.* [16]
15	Vulnibactin (vuuA) siderophore Aerobactin	Webster *et al.* [17]
16	*V. vulnificus* hemolysin (VvhA) Hemolysin	Jeong *et al.* [18]
17	Zinc protease	Miyoshi *et al.* [19]
18	Flp pilus	Gulig *et al.* [20]

## Discussion

*V. vulnificus* can be classified into three strains by biotyping [7]. Conventionally, Type 1 has been held to be pathogenic in humans, but recent studies have shown that Types 2 and 3 also cause infection in humans [7, 8]. Type 3 caused 62 cases in Israel in 1996–1997, all of which were considered to be wound infections associated with aquaculture of fish [7]. Reports of Type 3 *V* *. vulnificus* infection have been exclusive to Israel [9]. Rosche *et al.* [6] reported that strains of *V. vulnificus* can be classified into two genotypes: clinical type (C-type) and environmental type (E-type) according to gene sequences. In PCR analysis using prepared primers based on these genotypes, the C-type sequence was found in 90 % of strains of clinical isolates, and the E-type sequence in 93 % of strains of environmental isolates.

We presented a case of fulminant infection by *V. vulnificus* with Biotype 3 and Genotype E. This type of *V. vulnificus* had been believed to be a less severe pathological strain. Furthermore, this is the first case report in East Asia about Biotype 3 *V* *. vulnificus* infection, which has hitherto been reported only in the Middle East.

In conclusion, this case highlighted that even *V. vulnificus* of Biotype 3 or Genotype E thus far considered as being of low pathogenicity can cause primary septicaemia and suggested that the habitat of Biotype 3 strains has been extending to East Asia.

## Supplementary Data

246 Supplementary File 1
