# Supplementary material for: A case of Vibrio vulnificus infection complicated with fulminant purpura: gene and biotype analysis of the pathogen
Source: JMM Case Rep. 2017 May 19;4(5):e005096. doi: 10.1099/jmmcr.0.005096 (PMC5630965; doi:10.1099/jmmcr.0.005096)
Supplement: Supplementary File 1 [file jmmcr-4-5096-s001.pdf]

JMM CASE REPORTS

Translated Abstract for JMM Case Reports

Language: Japanese
Article Type: 症例報告
Title: 電撃性紫斑病を呈したVibrio vulnificus感染症の1例：病原菌の遺伝子型・生物型解析
Author/s: 堀雅俊1，中山章文2，北川大輔3，福島英賢1，川井廉之1，浅井英樹1，奥地一夫1
Affiliations: 1 奈良県立医科大学附属病院高度救命救急センター，2 岐阜医療科学大学保健科学部臨床検査学科，3 奈良県総合医療センター中央検査室
Correspondence email address: holy.ycher@gmail.com
DOI:

Abstract

背景：
Vibrio vulnificus（以下V. vulnificus）は易感染宿主において重症感染症を生じ得る。病型から1）原発性敗血症型，2）腸管感染型，3）創傷感染型の3つに分類されるが，このうち原発性敗血症型は致死的である。また，V. vulnificusは3つの生物型と2つの遺伝子型に分類され，それぞれの型によって病原性が異なるとされる。

症例：
症例は既往歴や海外渡航歴のない47歳の男性で，発熱，両下肢痛と下痢を主訴に近医を受診した。敗血症性ショックを呈しており，発症から24時間以内に電撃性紫斑病を伴った。高用量の血管収縮薬と抗生剤の投与を行ったが改善せず，第3病日に死亡した。血液，皮膚，鼻汁の培養からV. vulnificusが検出され，生物型解析と遺伝子解析を行った。生物型は主にイスラエルにおける創傷感染型として報告されていた3型で，遺伝子型は環境分離株に多いとされるE型であり，いずれも従来は病原性が低いとされたものであった。

結論：
自験例から，生物型3型や遺伝子型E型であっても原発性敗血症型を生じ得ることが示された。また，生物型3型は主に中東で報告されてきたが，東アジアまで分布域が拡大しているおそれがある。

Disclaimer: Translations of journal articles are provided by authors of those articles. No liability is assumed by *JMM Case Reports* or the Microbiology Society for any errors, omissions, or ambiguities in the translations provided. Any person or entity that relies on translated content does so at their own risk. *JMM Case Reports* or the Microbiology Society shall not be liable for any losses caused by reliance on the accuracy or reliability of translated information. If you would like to report a translation error or inaccuracy, please contact the editorial office.

MICROBIOLOGY SOCIETY
